# Supplementary material for: Parental Knowledge, Attitude, and Practices on Antibiotic Use for Childhood Upper Respiratory Tract Infections during COVID-19 Pandemic in Greece
Source: Antibiotics (Basel). 2021 Jul 1;10(7):802. doi: 10.3390/antibiotics10070802 (PMC8300644; doi:10.3390/antibiotics10070802)
Supplement: Supplementary file 1 [file antibiotics-10-00802-s001.zip › antibiotics-1268355-supplementary.pdf]

antibiotics

MDPI

Table S1. Association of demographic characteristics with knowledge statements about URTIs.

			p-value (chi-square test)										
**Statement**	**Correct Answer**	**Incorrect Answer**	**Gender**	**Age**	**Nationality**	**Education**	**Occupation**	**Insurance**	**Income**	**Number of Children**	**Age of Child**	**Child's Chronic Disease**	**Access to Health System**
Most infections with cold or flu symptoms are caused by viruses.	81.6%	17.4%	NS *	NS	0.000 I **: Other	0.000 I: Primary school	0.035 I: Part-time	0.000 I: None	0.005 I: Low	NS	NS	NS	0.008 I: Low
Antibiotics are needed for bacterial infec-tions.	48.5%	50.7%	NS	NS	0.029 I: Other	0.000 I: Primary school	0.001 I: Unem-ployed	0.022 I: None	0.000 I: Low	0.008 I: 4	0.000 I:12–16 years	NS	0.015 I: Good
Flu and com-mon cold need only sympto-matic treat-ment.	69%	28.7%	NS	0.000 I: 18–30 years	0.003 I: Other	NS	0.002 I: Students	NS	0.005 I: Low	0.016 I: 1	NS	NS	0.042 I: Very low
Antibiotic use may protect from complica-tions of com-mon cold or the flu.	48.2%	50.9%	NS	NS	0.010 I: Other	0.000 I: Primary School	0.035 I: Unem-ployed	NS	0.001 I: Low	0.049 I: 2	NS	NS	0.005 I: Very low
Antibiotics are always needed for my child's flu or common cold.	79.1%	19.9%	NS	0.000 I: 18–30 years	0.000 I: Other	0.000 I: Primary School	0.004 I: Unem-ployed	0.000 I: None	0.000 I: Low	0.037 I: 4	NS	NS	NS

Antibiotics are always needed for my child's tonsillitis.	19%	79.9%	NS	0.028 I: >60 years	NS	0.002 I: Primary School	NS	0.001 I: None	0.037 I: Low	0.001 I: 5	0.012 I: 12–16 years	NS	NS
Antibiotics are always needed for my child's otitis.	17.5%	81.1%	NS	NS	NS	0.003 I: Primary School	0.004 I: Students	0.000 I: None	0.000 I: Low	0.013 I: 5	NS	NS	NS
Imprudent use of antibiotics reduces their efficacy and may cause antibiotic resistance.	80.8%	17.7%	NS	0.000 I: >60 years	0.000 I: Other	0.000 I: High school	0.000 I: Part-time	NS	0.000 I: Low	NS	NS	NS	0.031 I: Low
My child will be sick for a longer time if it doesn't receive antibiotics for its cold or flu symptoms.	53.6%	45.4%	NS	NS	0.000 I: Other	0.001 I: Primary school	0.019 I: Part-time	0.011 I: None	0.000 I: Low	NS	NS	NS	NS

* NS: not statistically significant. ** For statistically significant associations for each statement the subgroup of parents who answered incorrectly (I) in a higher percentage are noticed in parentheses.

Table S2. Association of demographic characteristics with attitude/practice statements about URTIs.

			p-value (chi-square test)											
**Statement/Question**	**Correct**	**Incorrect**	**Gender**	**Age**	**Nationality**	**Education**	**Occupation**	**Insurance**	**Income**	**Number of Children**	**Age of Child**	**Single-Parent Family**	**Chronic Disease**	**Access to Health System**
I am more satisfied when my pediatrician prescribes antibiotics for my kid's cold or flu symptoms.	69.4%	30%	NS *	0.004<br>I **: 18–30 years	0.000<br>I: Other	0.000<br>I: High school	0.000<br>I: Unemployed	0.000<br>I: None	0.000<br>I: Low	0.018<br>I: 4	NS	NS<br>I: Yes	NS	0.032<br>I: Very low
I may visit another pediatrician for a second opinion if mine doesn't prescribe antibiotics	81.3%	18.3%	NS	0.001<br>I: 18–30 years	0.000<br>I: Other	0.000<br>I: Primary school	NS	NS	0.026<br>I: Low	NS	NS	NS	NS	0.002<br>I: Low
How often do you buy antibiotics at the pharmacy without medical prescription for your child's cold or flu symptoms?	85%	14.6%	NS	0.002<br>I: 51–60 years	0.003<br>I: Other	0.001<br>I: Primary school	NS	NS	NS	0.010<br>Λ: 4	NS	NS	0.023<br>Λ: Ναι	NS
How often do you insist your pediatrician to prescribe antibiotics for your child's cold or flu symptoms?	84%	15.5%	NS	0.000<br>I: 51–60 years	0.000<br>I: Other	0.000<br>I: Primary school	NS	NS	NS	NS	NS	NS	0.001<br>I: Yes	NS
How often does your pediatrician recommend antibiotics over the phone?	61.4%	38.1%	NS	NS	NS	0.022<br>I: Primary school	NS	NS	NS	NS	NS	NS	NS	NS

* NS: not statistically significant. ** For statistically significant associations for each statement the subgroup of parents who answered incorrectly (I) in a higher percentage are noticed in parentheses.
